# Interactions of TTV with BKV, CMV, EBV, and HHV-6A and their impact on post-transplant graft function in kidney transplant recipients

**DOI:** 10.3389/frtra.2024.1393838

**Published:** 2024-06-11

**Authors:** Kamil S. Rosiewicz, Arturo Blazquez-Navarro, Sviatlana Kaliszczyk, Chris Bauer, Michal Or-Guil, Richard Viebahn, Panagiota Zgoura, Petra Reinke, Toralf Roch, Christian Hugo, Timm Westhoff, Constantin Thieme, Ulrik Stervbo, Nina Babel

**Affiliations:** ^1^ Berlin Center for Advanced Therapies (BeCAT), Charité — Universitätsmedizin Berlin, Berlin, Germany; ^2^ Center for Translational Medicine, Universitätsklinikum der Ruhr-Universität Bochum, Medizinische Klinik I, Herne, Germany; ^3^ MicroDiscovery GmbH, Berlin, Germany; ^4^ Institute of Medical Immunology, Charité — Universitätsmedizin Berlin, Berlin, Germany; ^5^ Chirurgische Klinik, Universitätsklinikum Knappschaftskrankenhaus Bochum, Bochum, Germany; ^6^ Universitätsklinikum Carl Gustav Carus, Medizinische Klinik III — Bereich Nephrologie, Dresden, Germany

**Keywords:** TTV, KTx, BKV, CMV, Epstein–Barr virus (EBV), HHV-6A

## Abstract

**Background:**

Mono and combined reactivation of latent viruses occurs frequently under immunosuppressive therapy in kidney transplant patients. Recently, monitoring torque teno virus (TTV) reactivation came more into focus as a potential biomarker for immune status. The surrogate characteristics of TTV reactivation on acute rejection, and the combined reactivation with other latent viruses such as cytomegalovirus (CMV), human BK virus (BKV), Epstein–Barr virus (EBV), and human herpes virus-6A (HHV-6A) on allograft function, are unknown so far.

**Methods:**

Blood samples from 93 kidney transplant recipients obtained during the first post-transplant year were analyzed for TTV/BKV/CMV/EBV/HHV-6A load. Clinical characteristics, including graft function [glomerular filtration rate (GFR)], were collected in parallel.

**Results:**

TTV had the highest prevalence and viral loads at 100% and a mean of 5.72 copies/ml (cp/ml) (log_10_). We found 28.0%, 26.9%, 7.5%, and 51.6% of simultaneous reactivation of TTV with BKV, CMV, EBV, and HHV-6, respectively. These combined reactivations were not associated with a significantly reduced estimated GFR at month 12. Of interest, patients with lower TTV loads <5.0 cp/ml (log_10_) demonstrated not only a higher incidence of acute rejection, but also an unexpected significantly earlier occurrence and higher incidence of BKV and HHV-6A reactivation. Correlations between TTV loads, other latent viruses, and immunosuppressive medication were only significant from 6 months after transplant.

**Conclusion:**

We were able to observe and support previously introduced TTV load thresholds predicting kidney allograft rejection. However, due to a possible delayed relation between immunosuppressive medication and TTV viral load adaptation, the right time points to start using TTV as a biomarker might need to be further clarified by other and better designed studies.

## Introduction

Monitoring kidney transplanted (KTx) patients to prevent infections or rejection events are key goals in clinics. The identification of a reliable biomarker to track patients’ immune cell status is one of the big tasks of the current transplant research. So far, current methods include the monitoring of peripheral blood levels of immunosuppressive drugs or simply daily dose, which helps more to uncover drug related-toxicities then the over- or under-suppression of patients’ immune system function (1).

In 1997, a novel non-enveloped, circular, and single-stranded DNA virus was identified, the torque teno virus (TTV) (2). Later, with its non-pathogenic characteristics, and a prevalence of up to 90% in healthy and up to 100% in KTx patients, TTV came more into focus as a potential surrogate marker candidate for patients’ immune cell status (3–5). Recently, it was published that higher TTV viral loads were associated with a higher incidence of infections post transplant (6) and lower TTV viral loads with a higher proportion of rejection events in solid organ transplant (SOT) recipients (7). Opposing this, other reports suggested no correlation between TTV viral loads and other infections, such as the human BK virus (BKV) (8).

Reactivation of other latent viruses, such as BKV, Epstein–Barr virus (EBV), or cytomegalovirus (CMV), is known to increase the morbidity and mortality rate of KTx patients (9–12). Another virus, the human herpes virus-6 (HHV-6), has been observed to have a seropositivity of 96.4% in SOT recipients (13). In a previous study, we demonstrated the negative effect of combined CMV-BKV reactivation on renal function 1 year after transplant, even at low viral loads (14) The effect of TTV reactivation and its direct or indirect interaction with other latent viruses on the renal allograft function has not been described so far. The aim of this clinical study was to evaluate whether there are correlations between specific co-infections of TTV with BKV, CMV, EBV, or HHV-6A, whether these correlations are TTV viral load-specific (TTV high group vs. TTV low group), and whether these specific groups showed different outcomes on renal graft function [e.g., glomerular filtration rate (GFR)] during the first transplant year in KTx patients.

## Material and methods

### Study and patients

This non-interventional, prospective, multicenter, and investigator-initiated study was conducted to validate the biomarkers of the prospective randomized trial Harmony (NCT 00724022) (15). The study was carried out in compliance with the Declaration of Helsinki and Good Clinical Practice and was approved by the local ethics committee of the Charité-Universitätsmedizin Berlin (EA1/112/17). According to the Harmony trial, the inclusion criteria were a renal transplantation from an AB0 compatible donor with a negative crossmatch, a panel reactive antibody level less than or equal to 20%, and an age range of 18–75 years. The exclusion criteria were as follows: a second or third transplant, if the first was lost due to severe rejection within the first year; combined kidney transplantation with another organ; immunosuppressive therapy up to 6 months before transplantation; HIV positivity; leukopenia; and thrombocytopenia. Women of childbearing age must practice effective contraception. The characterization of the Harmony trial patient cohort was published by Thomusch et al. (15) and Blazquez-Navarro et al. (16).

In total, 93 patients met the inclusion criteria and were monitored over nine study visits during the first post-transplant year. Study visits were defined as day 0 (pre-transplant, d0), week 1 (wk1), week 2 (wk2), month 1 (m1), month 2 (m2), month 3 (m3), month 6 (m6), month 9 (m9), and month 12 (m12) after transplant.

### Immunosuppressive therapy

Patients were treated with a quadruple immunosuppressive therapy, including IL-2R antibody as the induction therapy and tacrolimus (TAC), mycophenolate mofetil (MMF), and steroids as the maintenance therapy. Patients who did not receive antiviral prophylaxis were monitored for CMV reactivation (pre-emptive approach). The duration of antiviral prophylaxis was 3 months.

### Monitoring of patients

Patients were monitored during all nine study visits at the local study centers. Thus, GFR, full blood count, and routine chemistry tests were recorded. The GFR is reported as estimated GFR (eGFR), applied by the chronic kidney disease - epidemiology collaboration (CKD-EPI) formula [ml min^−1^ 1.73 m^−2^ (17)].

### Quantification of viral loads

Peripheral blood samples were collected and analyzed for BKV, CMV, EBV, and HHV-6A using quantitative PCR (qPCR), as already reported (18). The quantification of serum TTV viral loads was performed by the commercially available qPCR kit TTV R-Gene® (Biomérieux, Ref. 423414) according to the manufacturer’s instructions.

### Statistical analysis

Continuous data between two groups were compared using the Mann–Whitney test or the Kruskal–Wallis test when comparing more than two groups. *P*-values were not corrected for multiple testing as this study was of an exploratory nature (19, 20). Cumulative incidences (rejection events, viral infections) were calculated and plotted using the Kaplan–Meier survival method and the comparison of survival curves was reported using the Log-rank (Mantel–Cox) test. Testing for Spearman’s correlation was done using R software (version 4.3.1) and the package Hmisc (version 5.1-1) executing the rcorr command. Categorical variables were tested using the chi-square test. A multiple linear regression model was calculated in R using the lm function. Here, data were also tested for homoscedasticity (bptest), autocorrelation (dwtest and bgtest), and multicollinearity (vif). The coeftest function was applied to correct for standard error and *p*-values. Other statistical analyses were carried out using GraphPad Prism (version 10.2.0).

## Results

### Kinetic of TTV load during the first post-transplant year

**Figure 1:**
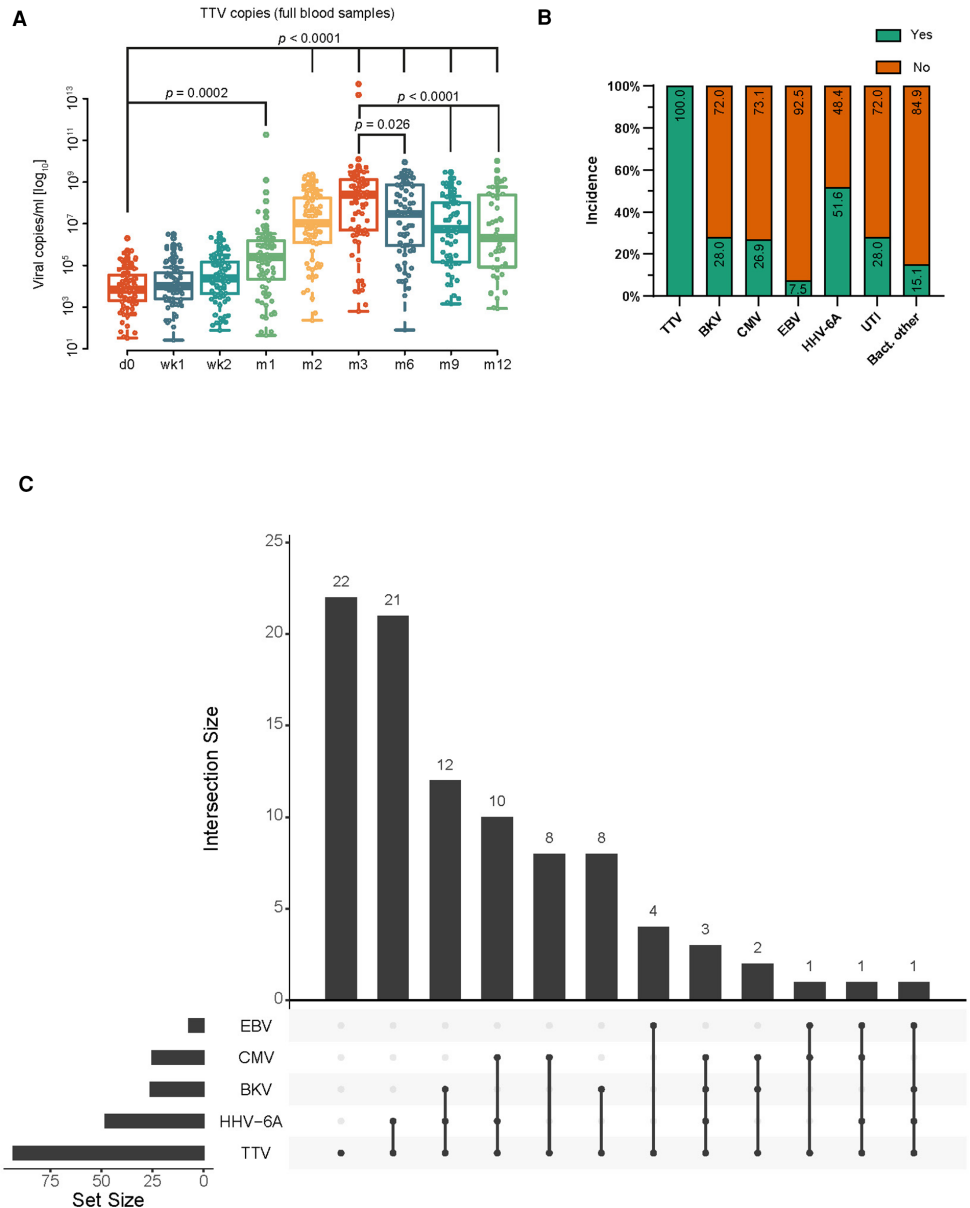
TTV kinetics and viral incidences. (**A**) TTV viral load over time. Please note that y-axis is log_10_ scaled. Time points on x-axis: d0 = pre-transplant; wk1–2 = post-transplant weeks 1–2; m1–12 = post-transplant months 1–12. (**B**) Incidence for TTV and other viruses/infections. (**C**) Visualization of all identified combinatorial co-infections. Numbers on top of bars symbolize the numbers of patients with specific viral co-infection. Calculated by R package UpSetR (27). Statistical analysis of TTV viral loads was carried out using the Kruskal–Wallis test.

In total, 93 KTx patients were enrolled in the present study. The basic characteristics of the study cohort are shown in Tables 1 and 2 and Supplementary Table S1. First, we analyzed the TTV loads of all patients at all nine visits using qPCR (shown in Figure 1A). The lowest mean viral loads of TTV were measured at both first visits, pre-transplant day 0 [4.2 copies/ml (cp/ml) (log_10_)] and post-transplant week 1 [4.1 cp/ml (log_10_)]. Then, a significant increase (*p* < 0.0001) in the TTV mean viral load was observed until post-transplant month 3, which also marked the visit with the highest TTV mean viral load [8.0 cp/ml (log_10_)]. Later, a significant decrease in the TTV mean viral load was detectable until post-transplant month 12 [6.5 cp/ml (log_10_), *p* < 0.0001]. Overall, the incidence of TTV was 100% (Figure 1B). In addition, we analyzed and evaluated the incidence of the following viral reactivations: BKV (28.0%, 26 patients); CMV (26.9%, 25 patients); EBV (7.5%, 7 patients); HHV-6A (51.6%, 48 patients); and urinary tract infection (UTI) (28.0%, 26 patients) and other bacterial infections (15.1%, 14 patients).

**Table 1:** Proportional characteristics based on sex of the study cohort.

Characteristic	Category	Males (*n* = 69)		Females (*n* = 24)		*χ* ^2^
		*N*	%	*N*	%	*p*-value
Rejection	Yes	15	21.74	2	8.33	0.143
	No	54	78.26	22	91.67	
BKV	Yes	20	28.99	6	25	0.708
	No	49	71.01	18	75	
CMV	Yes	17	24.64	8	33.33	0.408
	No	52	75.36	16	66.67	
EBV	Yes	5	7.25	2	9.09	0.777
	No	64	92.75	20	90.91	
HHV-6A	Yes	35	50.72	10	41.67	0.444
	No	34	49.28	14	58.33	
Type of donation	Living	9	13.04	1	4.17	0.227
	Deceased	60	86.96	23	95.83	
CMV risk	Yes	28	40.58	7	29.17	0.32
	No	41	59.42	17	70.83	
EBV risk	Yes	0	0	1	4.17	0.088
	No	69	100	23	95.83	
Antiviral prophylaxis	Yes	35	50.72	12	50	0.951
	No	34	49.28	12	50	
Graft loss	Yes	0	0	0	0	>0.9999
	No	69	100	24	100	
Previous transplants	Yes	4	5.8	2	8.33	0.663
	No	65	94.2	22	91.67	

Differences between male and female patients were tested using the chi-square test. CMV or EBV risk is defined as positive tested donor for CMV or EBV, while the recipient was negative for the respective virus.

**Table 2:** Clinical characteristics of the study cohort.

Characteristic	Males (*n* = 69)		Females (*n* = 24)		
	Mean	SD	Mean	SD	*p*-value
Age of recipient, years	54.55	12.71	55.42	16.00	0.557
Age of donor, years	55.28	12.48	53.71	15.42	0.815
CIT, min	606.50	273.10	644.40	287.70	0.849
MMF (mean), mg	1,754.00	243.80	1,772.00	272.10	0.648
TAC (mean), ng/ml	8.63	1.78	8.57	1.78	0.858
WBCs (mean), ×10^6^ cells/ml	8.14	2.19	8.00	2.16	0.811
TTV (mean), cp/ml (log_10_)	5.56	1.16	5.74	1.14	0.54
BKV load (mean), cp/ml	3.11 × 10^+5^	6.83 × 10^+5^	1.16 × 10^+5^	1.73 × 10^+5^	0.79
CMV load (mean), cp/ml	1.27 × 10^+4^	3.89 × 10^+4^	2.81 × 10^+4^	4.51 × 10^+4^	0.406
EBV load (mean), cp/ml	9.75 × 10^+2^	1.95 × 10^+3^	1.76 × 10^+2^	1.78 × 10^+2^	>0.9999
HHV-6A load (mean), cp/ml	1.89 × 10^+8^	5.07 × 10^+8^	6.67 × 10^+7^	2.11 × 10^+8^	0.262

Statistical comparison was performed sing the Mann–Whitney *U* test.

Next, we evaluated the incidence of patients with certain co-infections (Figure 1C). The most frequent co-infection was TTV + HHV-6A (48 patients) followed by TTV + BKV (26 patients), TTV + CMV (25 patients), and TTV + EBV (7 patients). Of note, 22 patients were negative for BKV, CMV, EBV, and HHV-6A. These patients will be defined below as TTV-only patients.

### Effect of TTV co-infection on post-transplant kidney function

**Figure 2:**
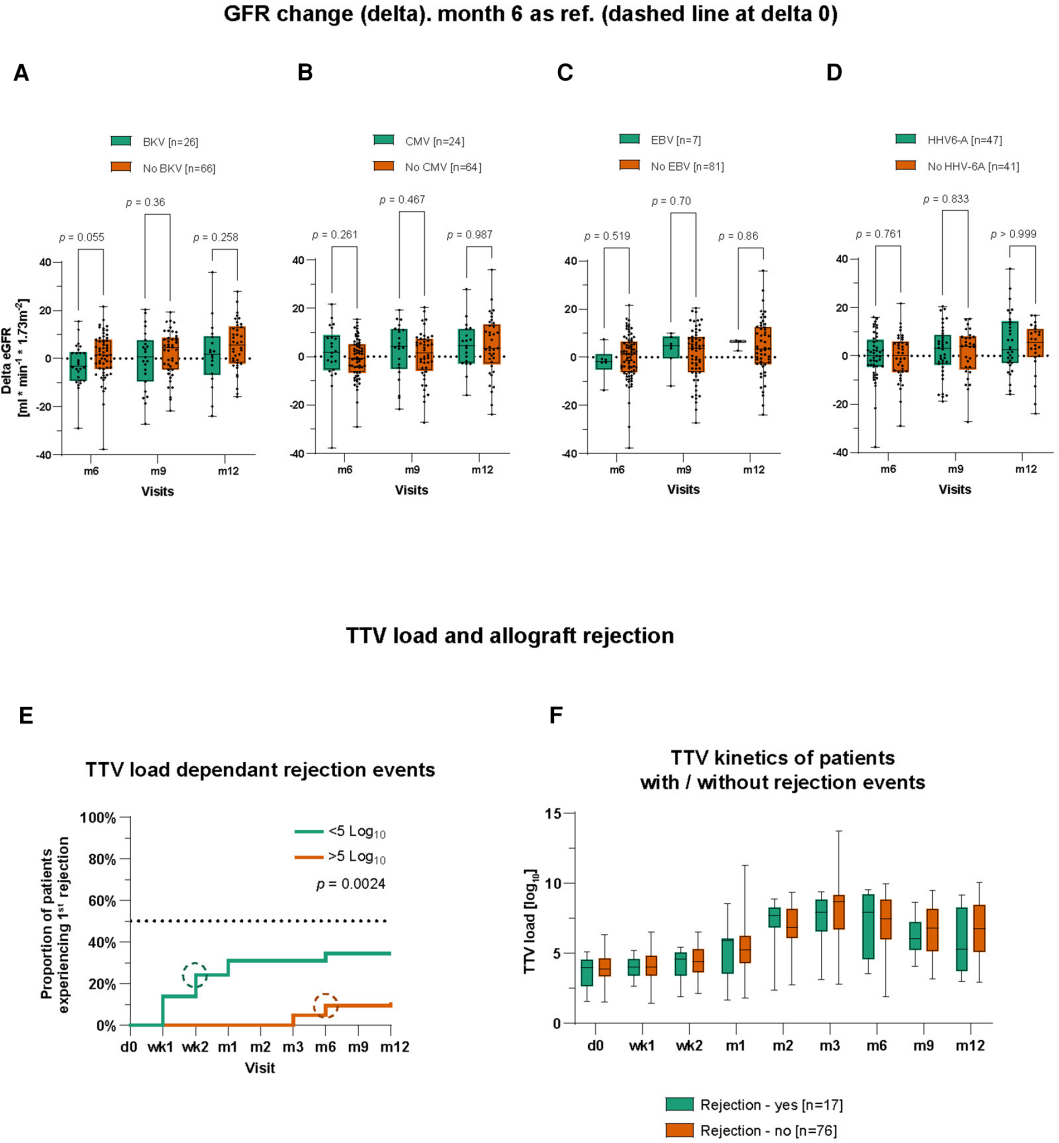
Influence of TTV co-infections on renal function and TTV load potentials as a biomarker predicting graft rejections. (**A**) eGFR was calculated and compared between BKV+ and BKV− patients. Month 3 was chosen as the reference for delta calculation. The same was done for CMV± patients (**B**), EBV± patients (**C**), and HHV-6A± patients (**D**). BKV+, CMV+, EBV+, and HHV-6A+ patients are not mutually exclusive; therefore, a patient might belong to more than just one group. (**E**) Kaplan–Meier curves to visualize the proportion of patients experienced a first graft rejection event during the first post-transplant year grouped by TTV viral load. Circles symbolize median time points. (**F**) Time course of TTV viral loads of patients with and without graft rejection. Time points on x-axis: d0 = pre-transplant; wk1–2 = post-transplant weeks 1–2; m1–12 = post-transplant months 1–12. Statistical comparison was carried out using the Mann–Whitney test (**A–D,F**) and Log-rank (Mantel–Cox) test (**E**).

To evaluate a potential impact on the kidney function driven by a certain co-infection, we analyzed the dynamics of renal function as defined by delta eGFR. The delta eGFR referred to a difference between eGFR of post-transplant month 3 and subsequent visits on months 6, 9, and 12. Although there was a visible tendency of a lower delta eGFR in BKV/TTV double-positive patients compared to BKV-negative patients, the difference was statistically not significant (Figure 2A). There was also no statistically significant difference between CMV/TTV, EBV/TTV, or HHV-6A/TTV double-positive patients compared to their CMV-, EBV-, or HHV-6A-negative group (Figures 2B–2D).

### TTV load as a biomarker of allograft rejection

Next, we wanted to evaluate TTV load as a potential biomarker for the immune cell status in KTx patients. According to the previously published data (21), we set a threshold of low and high TTV load as <5 cp/ml (log_10_) and >5 cp/ml (log_10_), respectively. Using this cutoff, we analyzed cumulative incidences for a first rejection event as well as for co-infections with BKV, HHV-6A, CMV, and EBV during the whole study observation period of 12 months in patients with high and low TTV load.

We started to compare the cumulative incidence of patients experiencing a first rejection event using the Kaplan–Meier survival curve. Patients with a low TTV load [<5.0 cp/ml (log_10_)] demonstrated a statistically earlier onset and higher incidence of acute rejection (in total, 34.5% vs. 10.9%) compared to patients with high TTV loads (median 1 month after transplant; *p* = 0.0024) (Figure 2E). Surprisingly, when comparing the kinetics of TTV measured by viral loads between patients with a rejection episode and patients without biopsy-proven rejections, there was no statistical difference during the first year after transplant (Figure 2F).

To exclude a possible bias by immunosuppressive regimen in patients with or without graft rejection, we compared the immunosuppressive medication (dose, through level) between the two groups. No clear difference was recorded regarding TAC blood levels or MMF and glucocorticoids dosing in patients with and without rejection events (Supplementary Figure S1). There was also no statistical difference in white blood cell (WBC) counts. In addition, we performed a correlation analysis and tested if patients’ mean TAC (blood levels), MMF (daily dosage), glucocorticoids (daily dosage), and WBCs showed a correlation to mean TTV viral loads. But, again, there was no significant correlation between the TTV viral load and TAC (*p* = 0.413), MMF (*p* = 0.098), glucocorticoids (*p* = 0.103), or WBCs (*p *= 0.482) (Supplementary Figure S2), when all data from the first year post transplant were analyzed. A multiple linear regression model was also applied to analyze the potential influence of several variables on the TTV viral loads. Among several independent variables such as age of recipient (*p* = 0.336), age of donor (*p* = 0.63), body mass index (BMI, *p* = 0.78), and cold ischemia time (CIT, min; *p* = 0.31), only HbA1c (%) values (*p* = 0.0001) showed a strong significant positive relation to TTV viral loads (Table 3).

**Table 3:** Multiple linear regression model to analyze confounder effects on the TTV viral load.

Variables	Estimate	Std. error	*p*-value
(Intercept)	0.0628	2.0480	0.0002
HbA1c	0.1867	0.2407	0.0001
Age of recipient	0.0581	0.0218	0.3362
Age of donor	−0.0292	0.0221	0.6265
BMI	0.0132	0.0434	0.7776
CIT, min	−0.0514	0.0009	0.3108

Results of the analysis for potential confounder effects of several independent variables on the TTV viral load (dependent variable). Analysis was performed in R software using lm function. TTV viral loads were previously log transformed. Data were tested for homoscedasticity (bptest), autocorrelation (dwtest and bgtest), and multicollinearity (vif). Standard errors and *p*-values were corrected using coeftest. Finally, all values were scaled to achieve a weighting of the independent variables on the TTV viral load (presented as “Estimate”). Multiple R^2^: 0.04055, adjusted R^2^: 0.02913, F-statistic: 3.55 on 5 and 420 DF, *p*-value: 0.003725.

### Increased incidence of BKV reactivation in patients with a low TTV load

**Figure 3:**
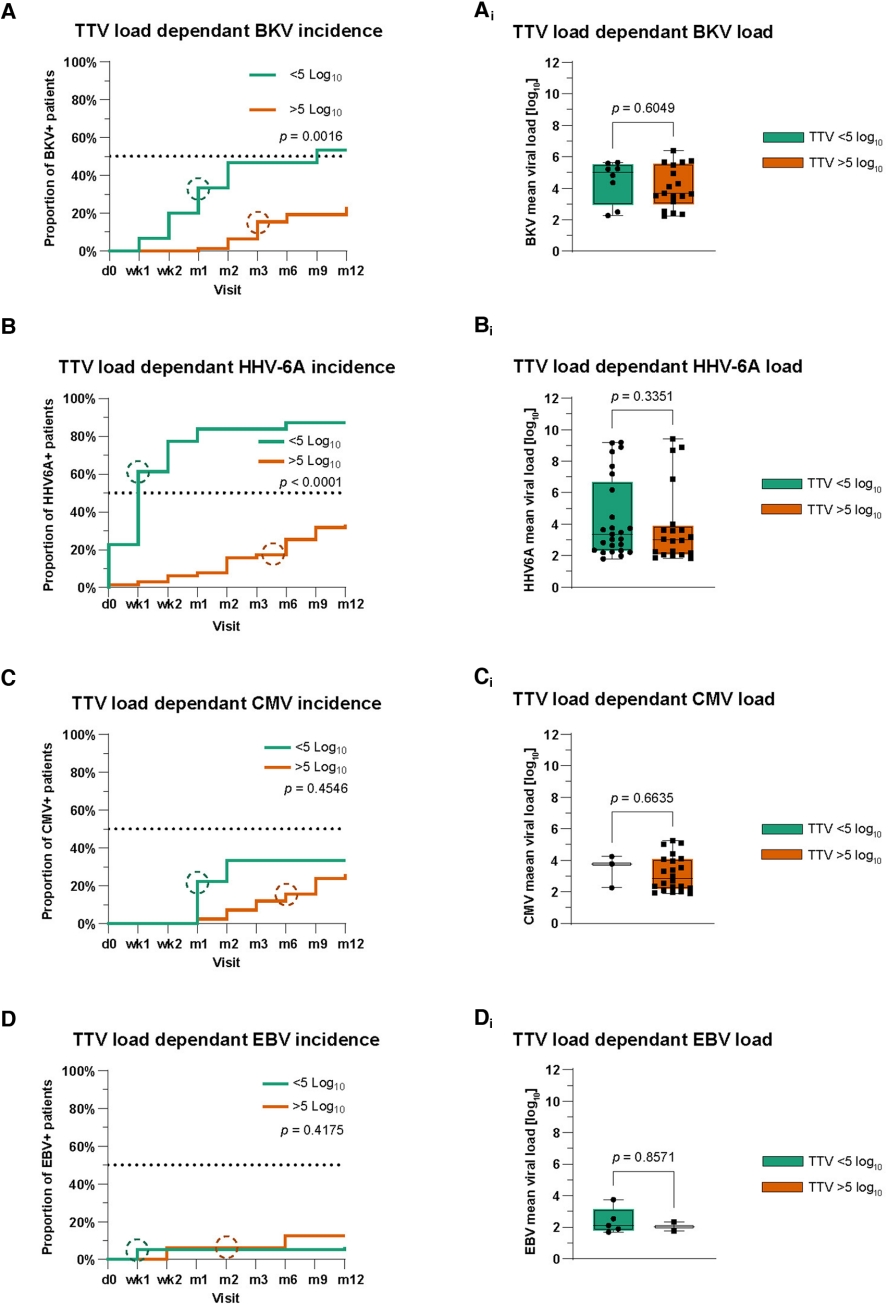
Analysis of TTV as a potential biomarker predicting viral reactivations. (**A–D**) Kaplan–Meier curves for the comparison of cumulative incidences with BKV (**A**), HHV-6A (**B**), CMV (**C**), and EBV (**D**). Circles symbolize median time points. Time points on x-axis: d0 = pre-transplant; wk1–2 = post-transplant weeks 1–2; m1–12 = post-transplant months 1–12. (**A_i_–D_i_**) Mean viral loads of BKV (**A_i_**), HHV-6A (**B_i_**), CMV (**C_i_**), and EBV (**D_i_**) during the first post-transplant year in patients based on TTV viral load. The Mann–Whitney test was used for the statistical comparison of mean viral loads.

We also wondered whether there is an association between TTV load and the kinetics of BKV, HHV-6A, CMV, and EBV reactivation. For this, patients were again divided into two groups based on their TTV loads measured before the reactivation of other viruses similar to the rejection episodes: patients with low TTV loads [<5.0 cp/ml (log_10_)] and patients with a high TTV loads [>5.0 cp/ml (log_10_)]. Of interest, the group of patients with lower TTV viral loads showed a significantly earlier occurrence and higher incidence of BKV reactivation (median 3 months post transplant; *p* = 0.0016) (Figure 3A). However, we did not find a statistical difference in BKV mean viral loads between the two TTV load groups during the whole study period (*p* = 0.605) (Figure 3A_i_).

Similar to BKV, we analyzed the association between TTV load and HHV-6A. We observed a significantly earlier and higher incidence of HHV-6A reactivation in patients with lower mean TTV loads [<5.0 cp/ml (log_10_)] compared to those of the high TTV load group (*p* < 0.0001) (Figure 3B). Similar to BKV, there was no difference in HHV-6A mean viral loads between TTV high and low groups during the whole study period (*p* = 0.34) (Figure 3B_i_).

Analyzing CMV and EBV in the same way, no statistical significance was detectable in patients in the low or high TTV load group (Figures 3C,D). Patients in both TTV viral load groups also showed no differences in their CMV and EBV mean viral loads (Figures 3C_i_,D_i_). In addition, we analyzed our data for differences in the cumulative incidence of urinary tract infections and other bacterial infections in patients in the low and high TTV groups. Again, we saw no statistical differences in the first post-transplant year (Supplementary Figures S3A,B).

### TTV viral load revealed potential biomarker properties starting 6 months post transplant

**Figure 4:**
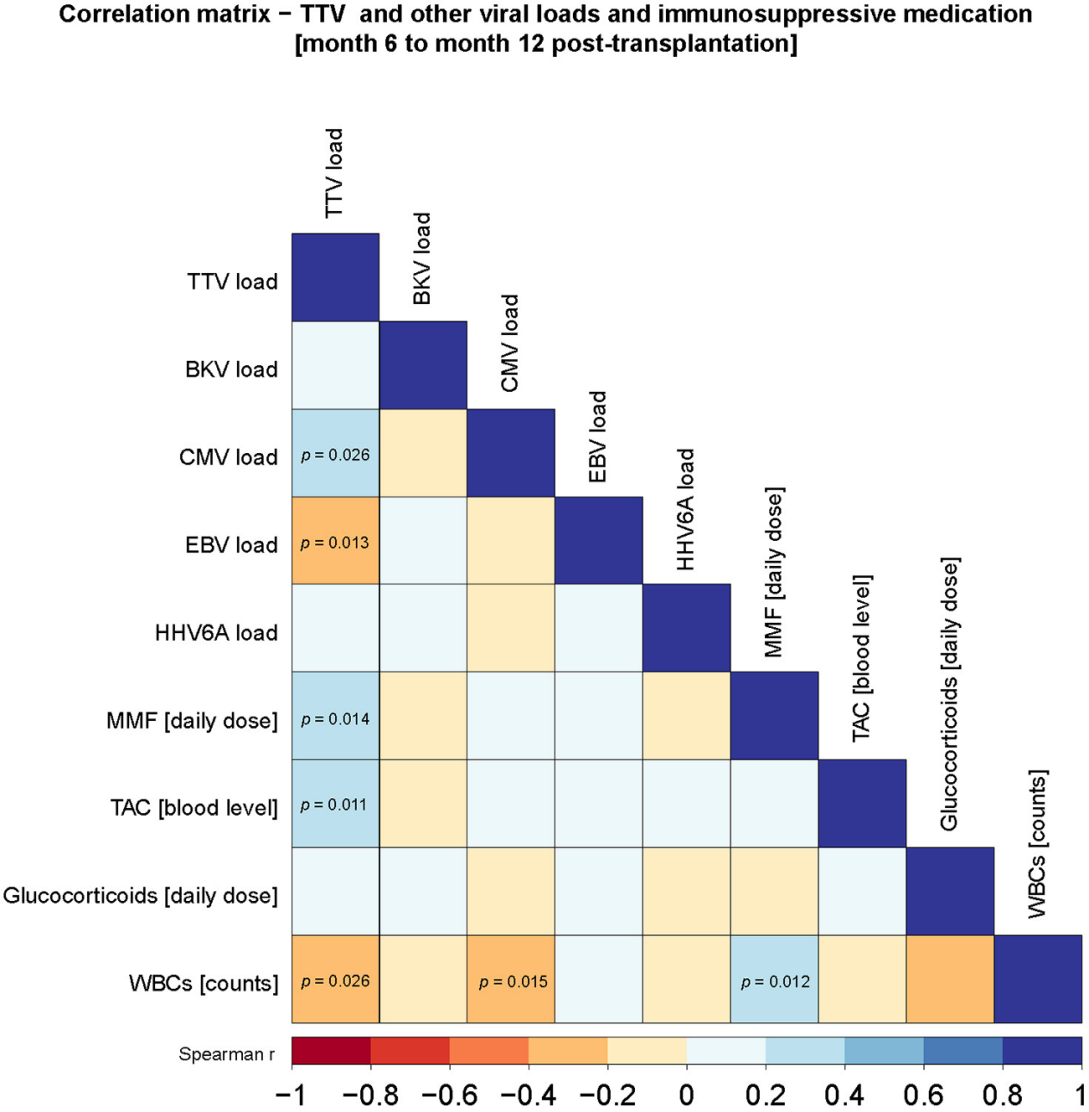
Correlation matrix of TTV and other viral loads and immunosuppressive regimen 6 months post transplant. Values collected during post-transplant months 6 and 12 showed a strong correlation to TTV viral loads. *p*-values are shown within the boxes. Color grading refers to Spearman’s rho rank correlation and is indicated in the legend underneath the correlation matrix.

Since Regele et al. (22) recently published a delay of up to 60 days between the change of immunosuppressive medication and a change in TTV viral loads, we re-evaluated our data considering the later time points, when immunosuppressive therapy achieves its stable maintenance regimen (from month 6). Surprisingly, we were able to detect several correlations between TTV mean viral loads and MMF daily dosage (*r* = 0.29; *p* = 0.014), TAC blood levels (*r* = 0.28; *p* = 0.011), WBCs (*r* = −0.24; *p* = 0.026), CMV (*r* = 0.24; *p* = 0.026), and EBV (*r* = −0.27; *p* = 0.013) mean viral loads (Figure 4). Interestingly, BKV (*p* = 0.68) and HHV-6A (*p* = 0.72) mean viral loads again showed no correlation to TTV mean viral loads. This result indicated that TTV might show reliable biomarker potentials at 6 months post transplant.

## Discussion

Preventing allograft rejection in KTx patients remains an essential task and balancing act for clinicians managing a sufficient drug-induced immunosuppression while preventing infection. The apathogenic TTV has been described to address immunocompetence and might be used as a potential candidate for immune monitoring (23).

In our current multicenter study, we evaluated the kinetics of TTV reactivation in 93 KTx patients and its potential interaction with BKV, CMV, EBV, and HHV-6A. TTV’s kinetics over a time frame of 12 months after transplant showed a characteristic course with slowly increasing viral loads until month 3 followed by decreasing viral loads until month 12. This is in line with other previously published reports (24, 25).

Using the previously established cutoff (7, 24, 25), we revealed that patients with a lower mean TTV load [<5.0 cp/ml (log_10_)] demonstrated a significantly earlier appearance and significantly higher incidence of acute rejection episodes compared to patients with a higher mean TTV load [>5.0 cp/ml (log10)]. Here, our data are in line with those of Strassl et al. (23), demonstrating TTV as a prospective biomarker for risk stratification of acute biopsy-proven alloreactivity in kidney transplant recipients. Interestingly, other studies have also reported that increased TTV viral loads were associated with a decreased risk for an allograft reaction without having an impact on other viral infections such as BKV and CMV (26). Therefore, the ongoing presence of graft rejection episodes in patients with higher mean TTV loads makes the handling and interpretation of the TTV viral load as a biomarker for a patient’s immunocompetence at least difficult. This is also reinforced by our additional result, that we could not detect a general difference neither in TTV viral loads nor in immunosuppressive regimen between patients with and without graft rejection episodes taking the whole year after transplantation into consideration.

Similar to acute rejection episodes, we used the mean TTV load cutoff measured before the reactivation of other viruses to address the role of TTV load as a biomarker for the reactivation of other latent viruses. We observed a significant earlier reactivation and a higher incidence of reactivation for BKV and HHV-6A in patients with lower mean TTV loads compared to those in the high mean TTV load group. No such differences were found between the low and high TTV groups for CMV and EBV considering the whole post-transplant year. The reasons for these contradictory results are so far not known and we could only speculate on these findings. A direct interaction between the viruses seems to be unlikely, since the viruses infect different targets. Some changes in immunosuppressive medication as a result of detected viral reactivation could at least partially explain our observation. Since the performed analyses are based on the multicenter study, center-specific variations in patient management cannot be completely excluded. Furthermore, co-reactivations of BKV/CMV or even BKV alone have been shown to have a negative impact on the renal function 1 year after transplant (14). In this study, we could not provide evidence that higher or lower TTV viral loads (<5 cp/ml [log_10_]) were associated with eGFRs in patients with co-reactivations of BKV, HHV-6A, CMV, or EBV (Supplementary Figures S4A–D).

Although further studies are required to confirm and address our observations regarding TTV viral load, graft rejection episodes, and other viral co-infections, Regele et al. (22) provided one plausible explanation for our contradictory results. They have shown that TTV viral loads adapt after just 60 days to a changed immunosuppressive regimen [calcineurin inhibitors (CNI)]. Therefore, our measured mean TTV loads and the interpreted patient’s immunocompetence could be delayed by approximately 2 months and therefore biased. Consequently, as an immunosuppressive regimen is under slow adjustment from the day of transplantation until the post-transplant months 1 and 2, a relation between drug-induced immunosuppression, TTV viral loads, and the risk for viral co-infections would be traceable just 2 or 3 months post transplant and not directly post transplant. This hypothesis is supported by our results, that only data of co-infection and immunosuppressive regimen showed a correlation to TTV’s viral load, when selectively analyzing data 6 months post transplant.

To exclude possible bias, we analyzed immunosuppressive therapy as well as other routinely collected clinical laboratory data. We could not detect any significant differences between the high and low TTV load groups regarding creatinine, albumin, cholesterol, calcium, and so on (data not shown).

Finally, we observed and supported previously introduced TTV load thresholds, which can be used to predict kidney allograft rejection during the first post-transplant year. However, our results do not support the boundless usage of monitoring TTV viral loads as a biomarker to predict hosts’ immunocompetence. With particular respect to the reactivation of other latent viruses, lower TTV loads within the first 2 months post transplant were associated with a higher incidence of BKV and HHV-6A reactivation. Therefore, we conclude that these initial lower TTV viral loads do not reflect precisely a patient’s immunocompetence. A significant and plausible correlation between TTV viral loads and other viral loads or a patient’s immunocompetence was only detectable 6 months post transplant. Combining our findings with those of other groups, we suggest intensifying the monitoring of TTV viral loads in KTx patients 6 months after transplant, as this period showed the most plausible usage of TTV as a biomarker for a patient’s immunocompetence and the consequence of a higher risk for co-infections. Further, better designed studies and those with a longer observation period (>1 year) are required to address our observation, including a potential relationship between HbA1c values and TTV viral loads.

## Data Availability

The raw data supporting the conclusions of this article will be made available by the authors, without undue reservation.
