# Supplementary material for: Interactions of TTV with BKV, CMV, EBV, and HHV-6A and their impact on post-transplant graft function in kidney transplant recipients
Source: Front Transplant. 2024 Jun 11;3:1393838. doi: 10.3389/frtra.2024.1393838 (PMC11235294; doi:10.3389/frtra.2024.1393838)
Supplement: Supplementary file 1 [file Table1.docx]

**Suppl Tab. 1: Type of biopsy proven kidney graft rejection and treatment**

| **Category** | **Type** | **n** | |
| --- | --- | --- | --- |
| 3 Borderline | Borderline | | 13 |
| 4 TCMR | IA | 4 | |
|  | IB | 3 | |
|  | IIB | 1 | |
| 2 AMR  +  4 TCMR | Active AMR  +  IIA | 1 | |
| **Treatments** |  | **n** | |
| Steroids |  | 15 | |
| Thymoglobulin |  | 4 | |
| Belatacept |  | 1 | |
| No treatment |  | 8 | |

Abbreviations: n = number of patients; TCMR = T cell-mediated rejection; AMR = antibody-mediated rejection.
