# Supplementary figures and images for: Interactions of TTV with BKV, CMV, EBV, and HHV-6A and their impact on post-transplant graft function in kidney transplant recipients
Source: Front Transplant. 2024 Jun 11;3:1393838. doi: 10.3389/frtra.2024.1393838 (PMC11235294; doi:10.3389/frtra.2024.1393838)

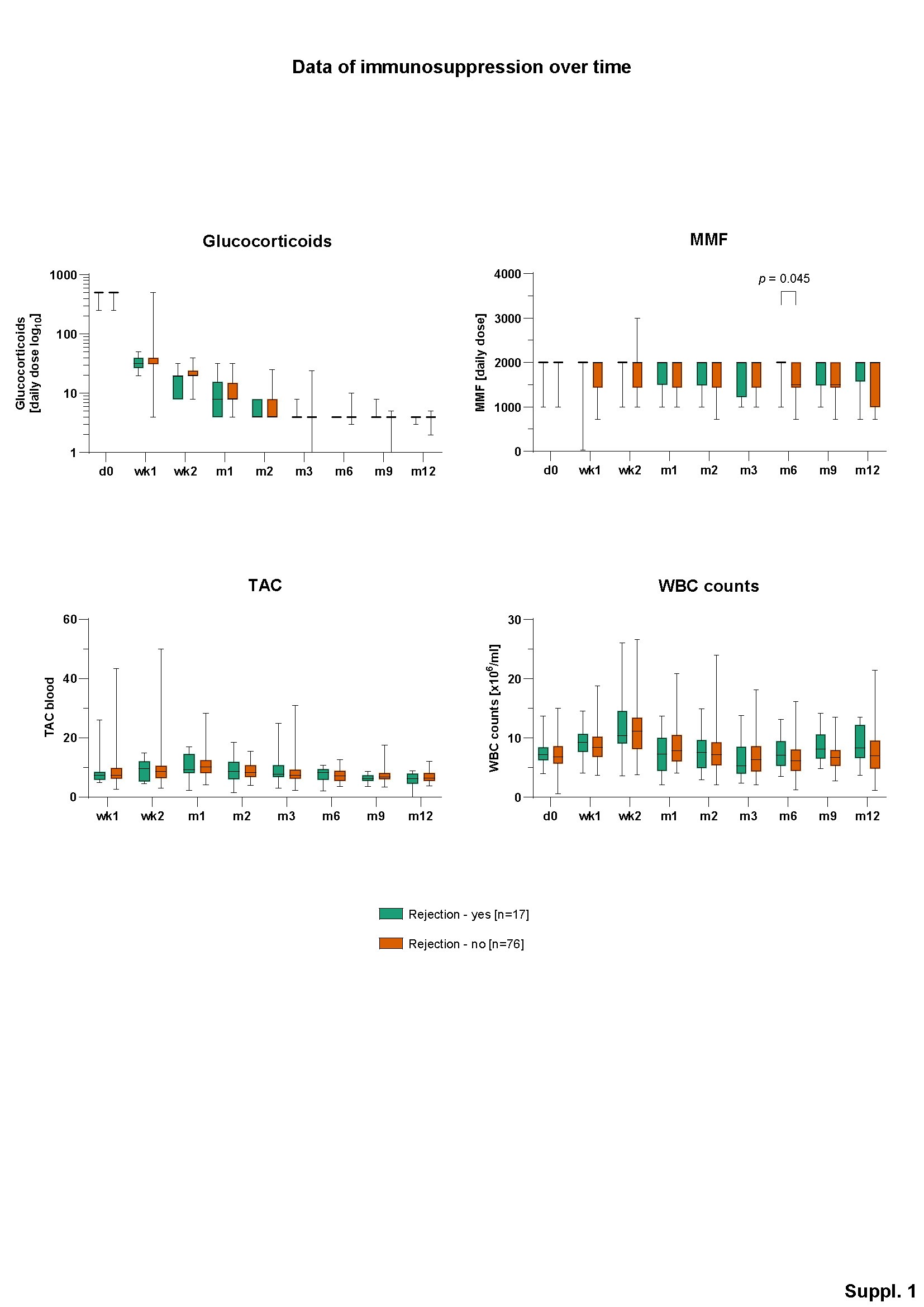

Supplement: Supplementary file 2 [file Image1.jpeg]

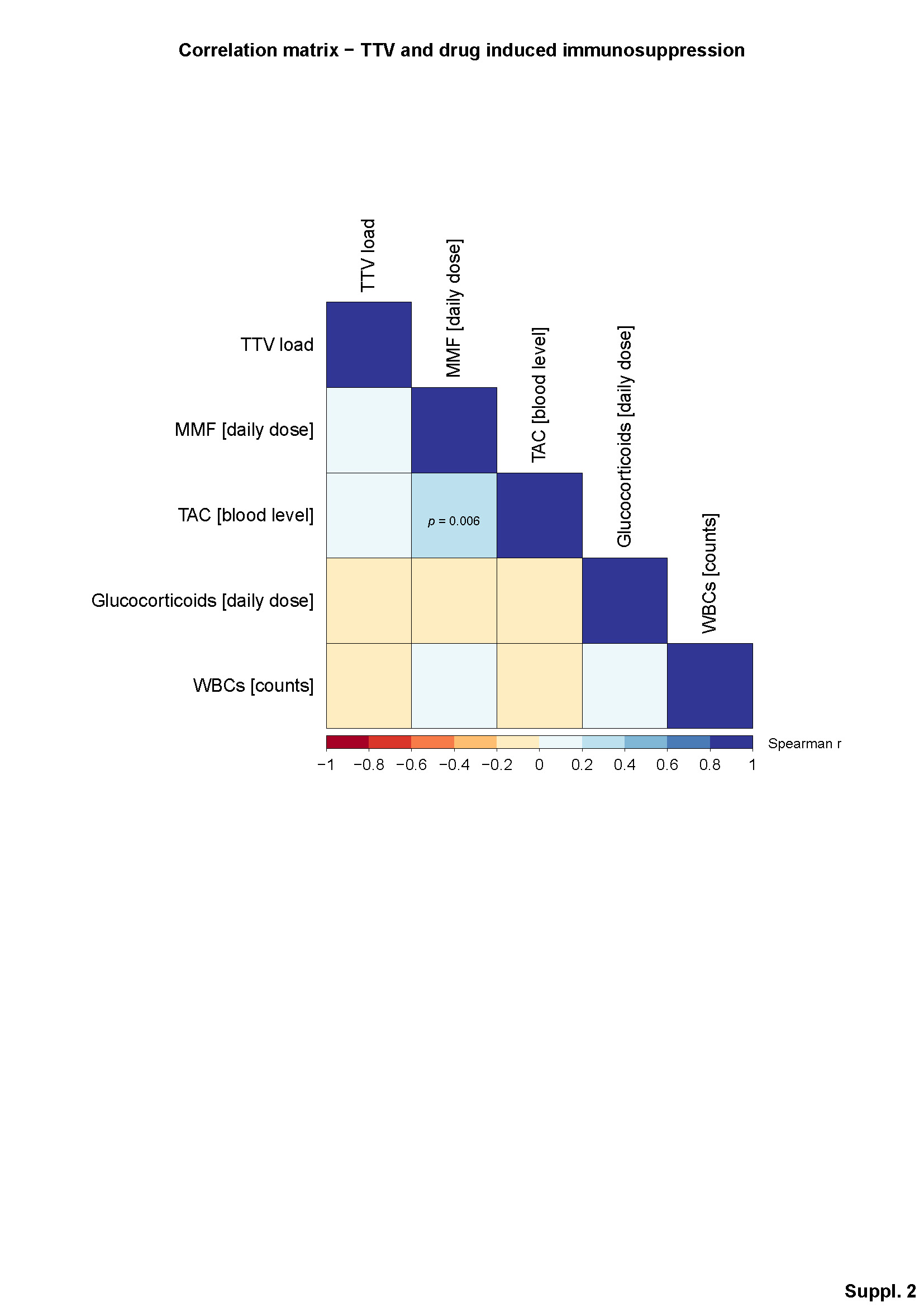

Supplement: Supplementary file 3 [file Image2.jpeg]

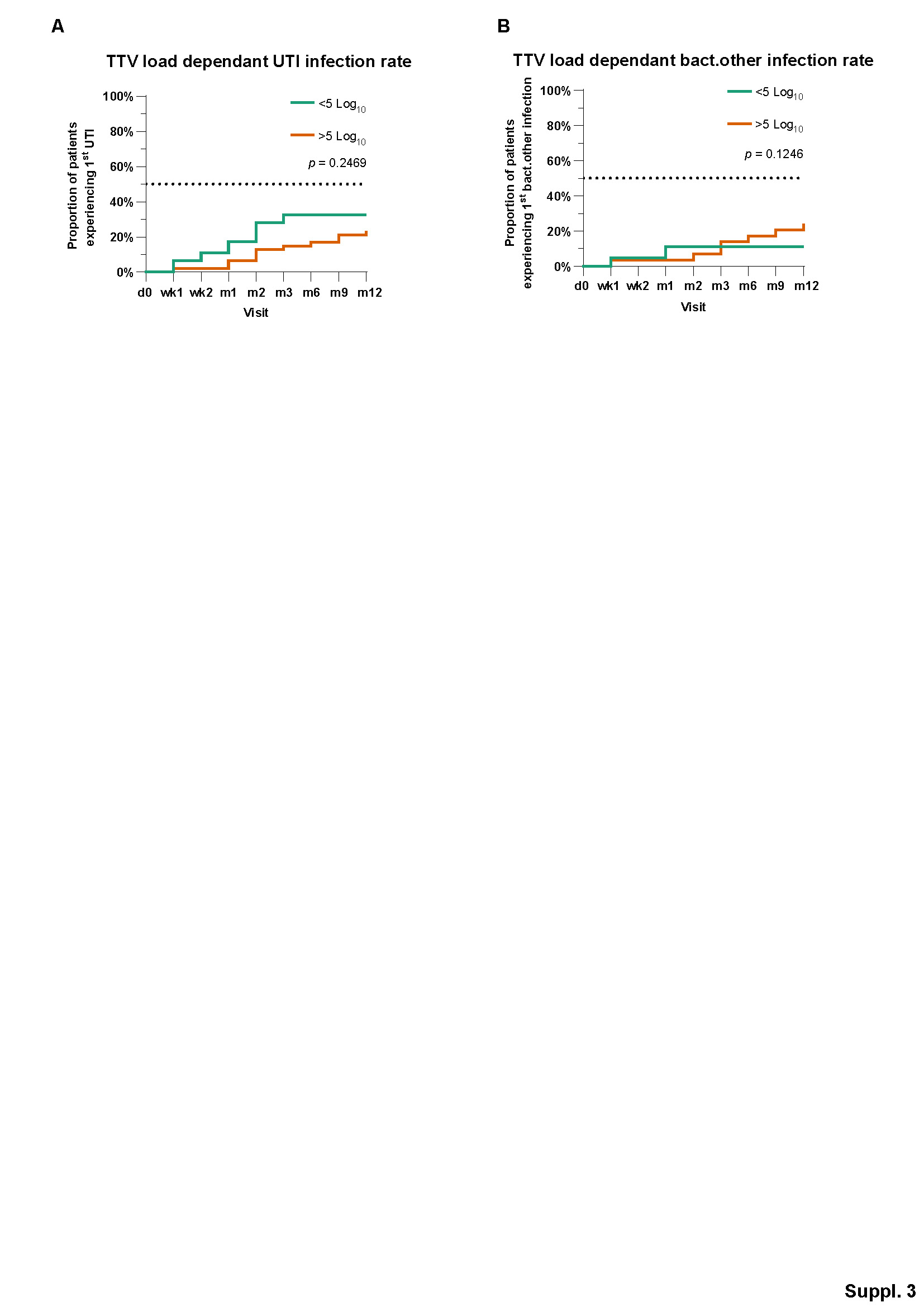

Supplement: Supplementary file 4 [file Image3.jpeg]

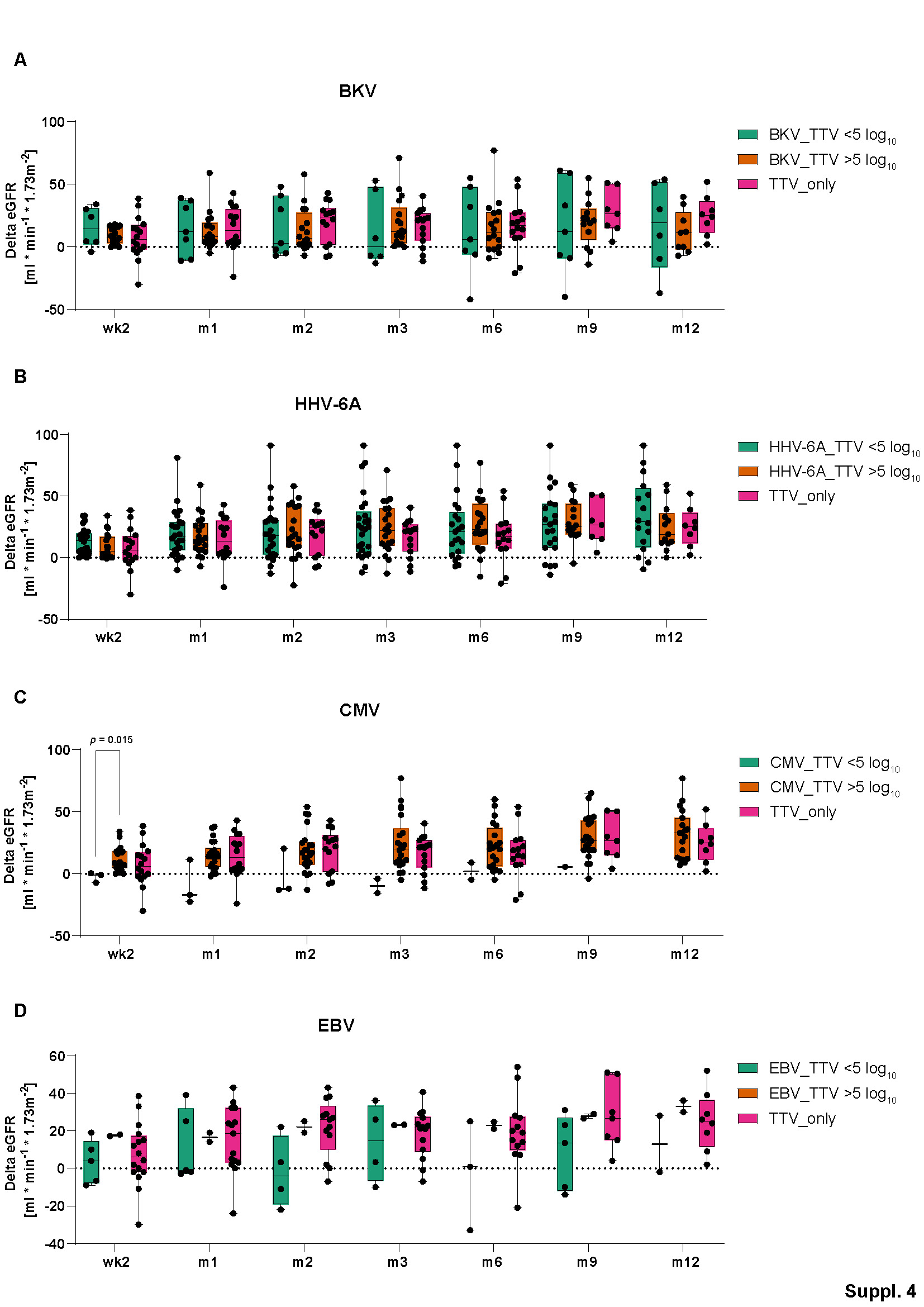

Supplement: Supplementary file 5 [file Image4.jpeg]
